# Well Controlling of Plasmonic Features of Gold Nanoparticles on Macro Porous Silicon Substrate by HF Acid Concentration

**DOI:** 10.1007/s11468-018-0720-8

**Published:** 2018-03-12

**Authors:** Alwan M. Alwan, Intisar A. Naseef, Amer B. Dheyab

**Affiliations:** 1 School of Applied Science, University of Technology, Baghdad, Iraq; 2 Ministry of Science and Technology, Baghdad, Iraq

**Keywords:** macroPSi/AuNPs, PECE, SERS active substrate, Cy molecules, Gold nanoparticles, Liquid detection

## Abstract

In this work, we fabricated an efficient macroporous silicon/gold nanoparticles (macro psi/AuNPs) hybrid structure and how well controlling of plasmonic features on macro psi/AuNPs employs them for highly sensitive detection of the very low concentration of cyanine (Cy) dyes molecules. Macro-PSi was synthesized on n-type Si wafer with 3–10 Ω. cm resistivity and 100 orientation using Photo Electro Chemical Etching (PECE) process with630 nm illumination wavelength and 30 mW/cm^2^ illumination intensity. The macroPSi /AuNPs hybrid structure substrates were prepared by simple and quick dipping process of macroPSi in tetrachloroauric gold solution HAuCl_4_ with different concentrations of (10^−2^  M, 10^−2^ M diluted in 2.9  M of HF, 5 × 10^−3^ M, and 5 × 10^−3^ M diluted in 2.9 M of HF). Efficient surface-enhanced Raman scattering (SERS) signals was obtained from macroPSi/AuNPs substrates for Cy dye concentration of about 10^−6^ and 10^−10^ M. The detection method is dependent on a nanoparticles sizes process through controlling the concentration in a HAuCl_4_ solution. Higher SERS signal was found for sample with lower salt concentration of 5 × 10^−3^ M diluted in HF. The enhancement factors (EF) of Raman’s signal increased four orders of magnitude by diluting the salt concentration. The values of EF in the range of 0.8 × 10^3−^0.72 × 10^7^ were obtained by controlling the salt concentration from 10^−2^ to 5 × 10^−3^ diluted in HF acid.

## Introduction

Porous silicon (PSi) is a sponge-like network of crystalline silicon with pillars and nodules of nanometer dimensions [1]. The main feature of detecting a medium for delicate sensing of the chemical analytics is the surface properties of itself material. Large surface area, porosity, topography, morphology, and surface functionality influence the identifying abilities to sense medium and its interaction with the adsorbate [2]. For these reasons, the use of PS is increasing in technological applications like the detection process of gas vapors and chemical dissolved materials. The physical properties of PS varied according to the shape, diameter of pores, porosity, and the porous thickness [3].

The chemical treatment of the porous surface by a reduction process through the dangling bonds with Au ion represents an important potential of surface-modified process to form efficient metal nanoparticle PS hybrid structure [1]. The SERS effect of the hybrid structure represents one of the most important techniques to the chemical detection process [4]. Gold nanoparticles (AuNPs) in the diameter range 1–100 nm present significant study topics in nanotechnology due to their profound properties, including the large surface to volume ratio and exceptional optical and plasmonic features (hot spots and localized surface plasmon). These features depend significantly on the size and shape of nanoparticles, in addition to gap among the deposited [5]. Cyanine dyes have high chemical and photochemical reactivity [6]. This material is very dangerous and has negative effects on health, which may lead to sudden death in the overdose case [7]. The silver nanoparticles/PS hybrid structure was studied by F. Giorgi’s et al. [8]; they found that the very low concentrations of Cy molecules from 10^−7^ to 10^−8^ M. The effectiveness of the substrate is firmly associated with the morphology of silver nanoparticles, which is deposited at different times.

Alessandro Virga et al. [9] have employed silver nanoparticles created inside a meso PS medium for Cy molecule’s detection process. Enhancement factors larger than 10^10^ was obtained via tuning the particle plasmonic resonance to be close to the molecule electronic resonance.

In this work, we have made extensive studies of the effect of gold nanoparticle size, distribution, and morphology on the activity of SERS substrates for efficient detection process of Cy molecules. The obtained results were discussed and analyzed.

## Experimental

### Chemicals and Materials

Hydrofluoric acid 24% (CDH), India, was used and diluted with high purity ethanol 99.8% (Sigma-Aldrich, Germany) to prepare the required concentration for the etching solutions of about 24% HF. HAuCl_4_ (Aldrich, 99.99%) and their mixtures with HF acid of about 10^−2^ M, 10^−2^ M diluted in 2.9 M of HF, 5 × 10^−3^ M, and 5 × 10^−3^ M diluted in 2.9 M of HF. The concentration of the dye and HAuCl_4_ solution was calculated by [10]:

1 $$ \mathrm{Molarity}=\frac{\frac{\mathrm{W}}{\mathrm{M}.\mathrm{Wt}}}{\mathrm{V}} $$

where W is the weight to the material in grams, M.Wt. is the molecular weight in (gm/mole), and V is the volume of the solution.

Organic cyanine (Cy) dye used in our study was purchased from GE Healthcare Life Science. The solid powder was dissolved in absolute ethanol to prepare 10^−4^, 10^−6^, and 10^−10^ to dye concentrations and used to investigate the SERS response of the prepared PS/AuNPs hybrid structure substrates. PS samples were prepared by a photoelectrochemical etching (PECE) process at room temperature of n-type 100 oriented Si substrates with a resistivity of 3–10 Ω cm. The Si substrates have been divided into pieces of 1.5 × 1.5 cm^2^ areas and then washed by HF: ethanol mixture at 1:10 to remove the native oxide layer from them. The etching process was carried out in 1:1 mixture of 48% of hydrofluoric acid (HF) of concentration 24 and 99% ethanol (C_2_H_5_OH (for 16-min etching time and a current density of about 10 mA/cm^2^. Laser radiation of wavelength 630 nm and illumination intensity of about 30 mW/cm^2^ was focused on silicon surface of about 1 cm^2^. The porosity and layer thickness have been calculated using a gravimetric method [11]. The values of porosity and layer thickness were about 74 and 38.4 μm, respectively.

### Synthesis of PS/AuNPs Hybrid Structures

The hybrid structures were prepared by passivation of the gold nanoparticles on PS surface by reduction of gold ions to gold nanoparticles (AuNPs) using a simple and quick immersion process. Different concentrations of (HAuCl_4_) solution with HF additive acid (10^−2^ M, 10^−2^ M diluted in 2.9 M of HF, 5 × 10^−3^ M, and 5 × 10^−3^ M diluted in 2.9 M of HF) were employed under dark conditions at a fixed deposition time of 3 min. For SERS activity measurements, the prepared hybrid structures of PS/AuNPs samples were incubated in Cy dye dissolved in ethanol with different concentrations 10^−4^, 10^−6^, and 10^−10^ M at a fixed dipping time of 15 min. The reduction process of AuNPs by the dangling bonds of the porous layer is given by the following equations [12]:

2 $$ 2\mathrm{Si}+6\mathrm{HF}+{\mathrm{H}}_2{\mathrm{SiF}}_6+\mathrm{H}+4\mathrm{e} $$

3 $$ {\mathrm{Au}}^{+3}+{\mathrm{e}}^{-}\to \mathrm{Au} $$

### Characterization

The structural properties of bare PS and PS/AuNPs hybrid substrates were investigated, using (XRD 6000 Shimadzu) and scanning electron microscopy SEM (AIS2300C). The SERS spectra was measured by (RENISHAW) via micro-Raman spectroscopy system using 514-nm radiation from an argon laser with an incident laser power of about 5 mW as the excitation source. The ImageJ program was used to calculate the statistical distribution of pores over sizes based on SEM images.

## Results and Discussion

### XRD Measurements

**Fig. 1:**
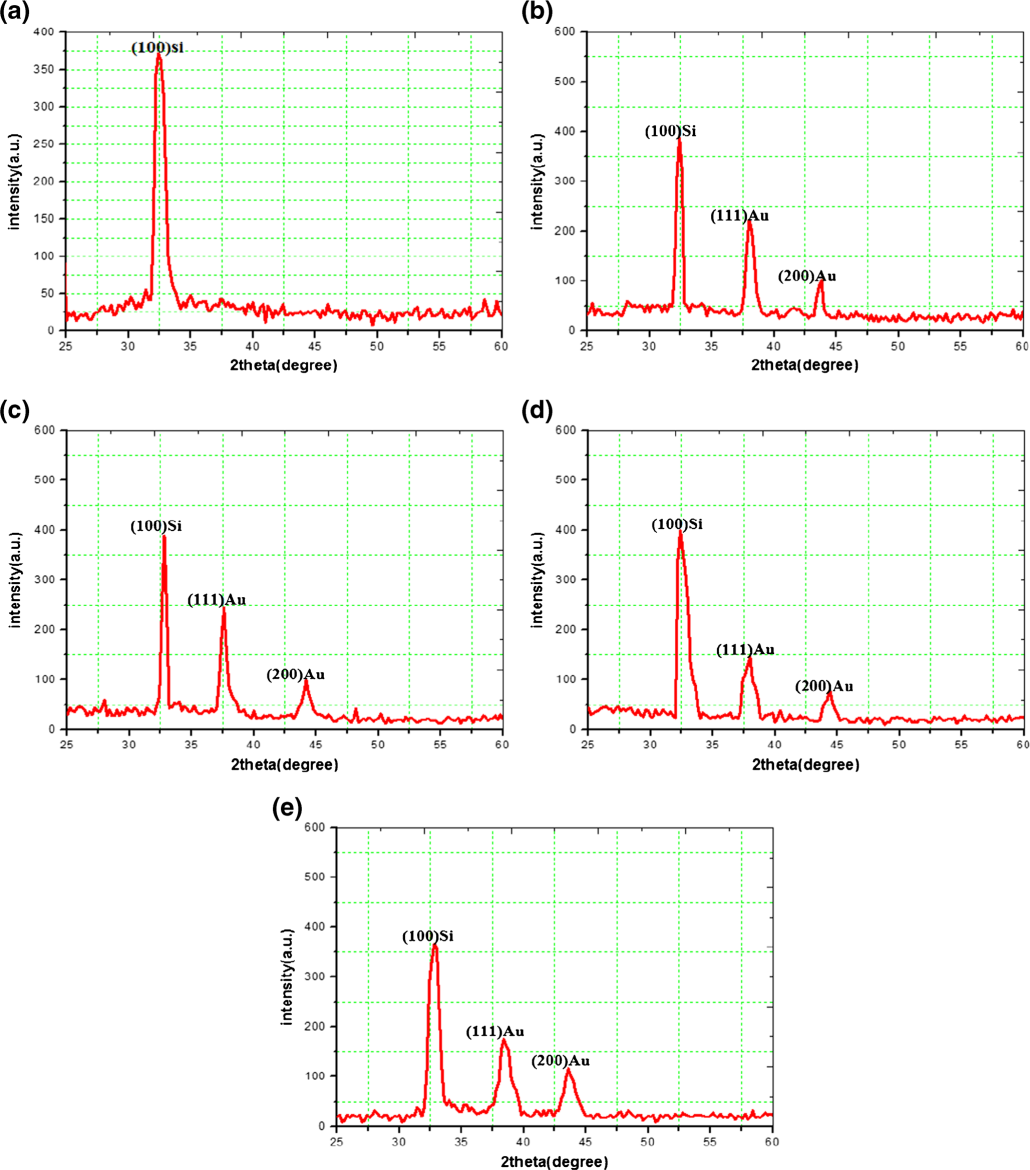
The XRD spectra for (**a**) bare PS (**b**, **c**, **d**, **e**) PS/AuNPs hybrid structure samples deposited with HAuCl_4_ at concentrations of 10^−2^ M, 10^−2^ M diluted in HF, 5 × 10^−3^ M, and 5 × 10^−3^ M diluted in HF, respectively

**Fig. 2:**
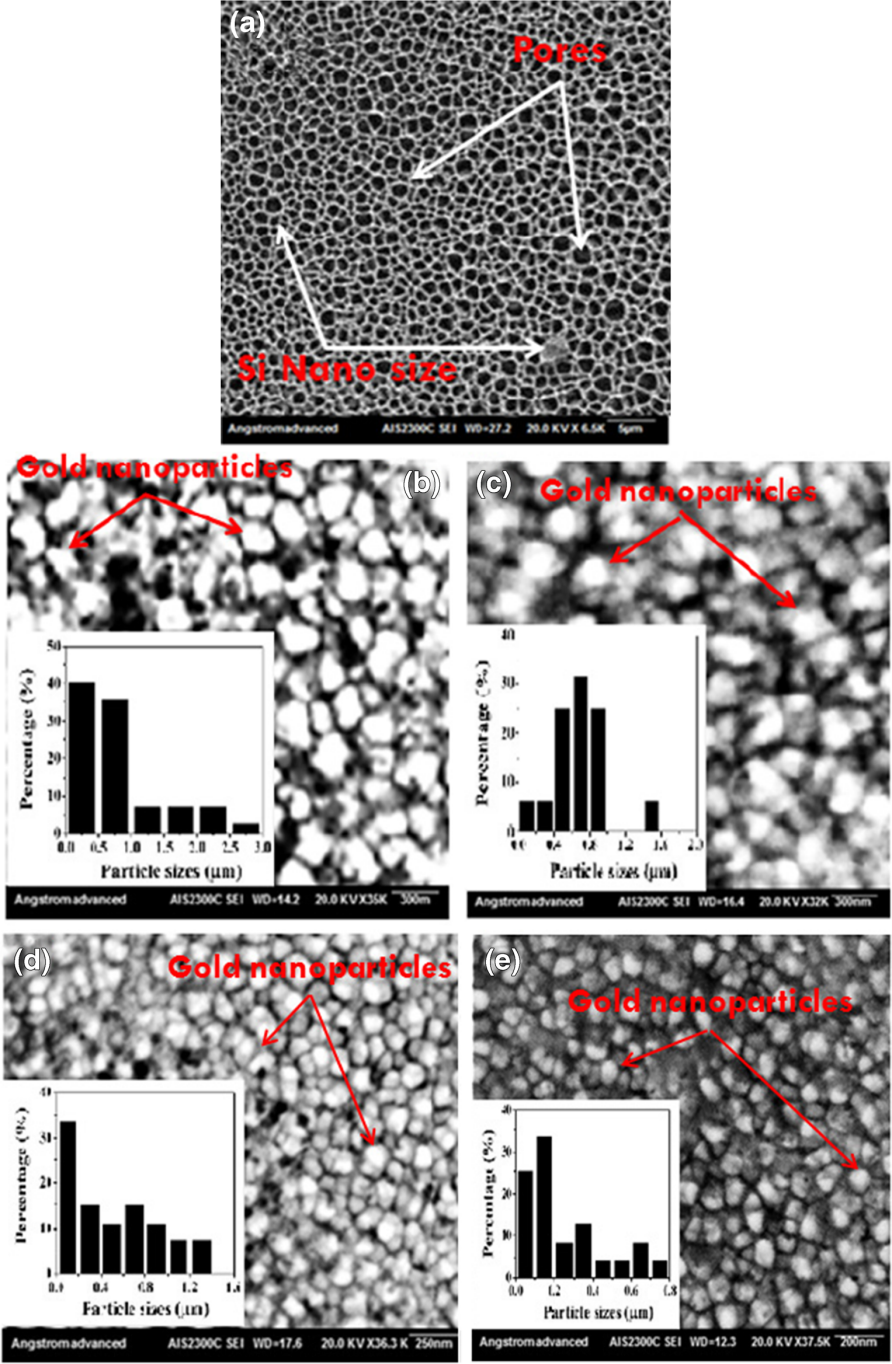
The SEM images of (**a**) bare PS (**b**, **c** ,**d** ,**e**) PS/AuNPs hybrid structure samples deposited with HAuCl_4_ with concentrations of 10^−2^ M, 10^−2^ M diluted in HF, 5 × 10^−3^ M, and 5 × 10^−3^ M diluted in HF, respectively

Figure 1 illustrates the XRD analysis of bare PS and PS/AuNPs hybrid structure samples made by deposition of AuNPs on PS sample at different concentrations of HAuCl_4_. From Fig. 1a, the bare PS layer stills crystalline along 100 plane at a diffraction angle of about 32.4^°^, while for Fig. 2b–e) present the XRD measurements of PS/AuNPs hybrid structure samples deposited with different concentrations of HAuCl_4_. Figure 1b presents the XRD spectra for PS/AuNPs hybrid structure at HAuCl_4_ concentration of 10^−2^ M giving the specific Bragg’s reflections at diffraction angles of 38^°^ and 44.4^°^ for the planes 111 and 200, respectively. Figure 1c illustrates the XRD spectra for PS/AuNPs hybrid structure with HAuCl_4_ concentration of about 10^−2^ diluted in 2.9 M of HF that giving the specific Bragg’s reflections at diffraction angles of 37.6^°^ and 44.2^°^ for the planes 111 and 200, respectively. Figure 1d demonstrates the XRD spectra of hybrid structure PS deposited with HAuCl_4_ at a concentration 5 × 10^−3^ M, diffraction peaks at 38^°^ and 43.8 corresponding to 111 and 200 planes, respectively. Finally, Fig. 1 presents the XRD spectra of hybrid structure PS deposited with HAuCl_4_ at a concentration 5 × 10^−3^ M diluted in 2.9 M of HF. The reflection planes were noticed at the 37.8° and 44°corresponding to 111 and 200 planes, respectively. The nanocrystallite sizes of gold were calculated from the peak broadening as shown in and can be obtained by using Scherer’s formula as follows [13]:

4 $$ \mathrm{D}=0.89\lambda /\left(\beta COS\ \theta \right) $$

D is the nanocrystallite size for PS layer in (nm), *λ* is the wave length in (nm) of employed radiation, *β* (in radians) is the full width at half maximum (FWHM), *θ* (in radians) is the diffraction angle, and 0.89 is the shape factor value.

The specific surface area (S.S.A.) is one of the figures of the merit of material. It is the surface area per mass and given as [13]:

5 $$ S.S.A.=\frac{6000}{D\ast \rho } $$

*D* is the size of the particles, and *ρ* is the density of gold 19.3 gm/cm^3^.

From Fig. 1, it is clearly seen that there is a slight shift in diffraction angles (2θ) or interaction space; this change is due to the presence of local microscopic deformation strain, i.e., the local variation of interatomic space of hybrid structure PS samples [14]. Table 1 lists the values of AuNPs sizes, full-width half maximum (FWHM), and specific surface area S.S.A. for AuNPs.

**Table 1:** Grain size, FWHM, and specific surface area S.S.A. values of AuNPs synthesized at different concentrations of HAuCl_4_ solution

HAuCl_4_ concentration (M)	Plane (111)			Plane (200)		
	FWHM (rad)	Size of AuNPs (nm)	S.S.A. of AuNPs (m^2^/gm)	FWHM (rad)	Size of AuNPs (nm)	S.S.A. of AuNPs (m^2^/gm)
10^−2^	0.007	20.94	14.84	0.014	10.69	29.08
10^−2^ + HF	0.01	14.64	21.23	0.017	8.8	35.32
5 × 10^−3^	0.02	7.32	42.47	0.018	8.29	37.50
5 × 10^−3^ + HF	0.028	5.23	59.44	0.021	7.11	43.72

Table 1 shows that the maximum gold grain size is about 10 nm for salt concentration of about 10^−2^ M. In the plane (200), the minimum gold grain size is about 6.6 nm for the HAuCl_4_ salt concentration of about 5 × 10^−3^ M diluted in 2.9 M of HF for the same plane. The highest value of S.S.A for AuNPs is about 47 m^2^/gm at HAuCl_4_ salt concentration of about 5 × 10^−3^ and the lowest value is about 31 m^2^/gm.

The decreasing of gold grain sizes and increasing of S.S.A. for AuNPs can be explained on the basis that the gold formation process through the ion reduction mechanisms depends on HAuCl_4_ concentration before and after adding HF acid.

The influence of the HF additive to the dipping solution is to control on the formation of gold nanoparticles, where the HF acid plays a double role; it contributes to promoting the adhesion of AuNPs onto the pores of Si substrate. On the other hand, it destabilizes and reduces the ability of aggregation of AuNPs and leads to small synthesis sizes. This result is in good agreement as compared with those results of [1].

### Surface Topography Measurements:

The topographical characteristics of bare PS like pore size, pore shape, and pore distribution and hybrid structure PS like AuNPs size, form, and distribution were investigated via the SEM analysis. This analysis showed the complex structures of bare PS surface due to the existence of randomly distributed pores. The diameter of the pores is ranging from 0.5 to 8 μm, and the distribution peak is about 0.7 μm. The SEM images of hybrid structure PS/AuNPs showed different sizes and morphologies of AuNPs and hot spot regions among the gold nanoparticles as a function of the concentration of HAuCl_4_ deposition solution. Figure 2 illustrates the surface morphology of bare PS and hybrid structure PS deposited with HAuCl_4_ solution of different concentrations 10^−2^ M, 10^−2^ M diluted in 2.9 M of HF, 5 × 10^−3^ M, and 5 × 10^−3^ M diluted in 2.9 M of HF.

The SEM images showed that the growth of AuNPs took place due to the high nucleation rate and the fast growth of AuNPs on the density of nucleation sites (Si-H_x_) (dangling bonds) that provide large and packed AuNPs located densely on the porous surface rather than inside the pore itself. The main reason for such fast nucleation and growth of closed packed regions is the high value of electronegativity difference between Au and Si, and AuNPs will grow in an isotropic way depending on the amount of the (Au^+3^) ions and the density ion reduction sites. The gold aggregation process on the porous surface was decreased by adding HF acid to the solution.

**Fig. 3:**
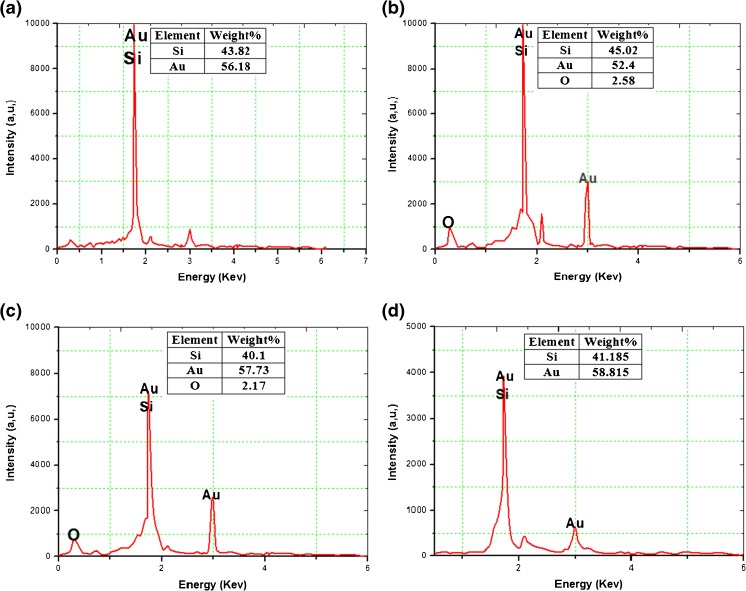
The EDS analysis of PS/AuNPs hybrid structure deposited with HAuCl_4_ with concentration of **a** 10^−2^ M. **b** 10^−2^ M diluted in HF. **c** 5 × 10^−3^ M. **d** 5 × 10^−3^ M diluted in HF

The statistical distribution of PS/AuNPs in hybrid structure substrates was varied with the salt concentration and solution composition. Figure 2b illustrates the statistical distribution of the AuNPs which ranges from 0.25 to 3 μm, and the peak of the size is around 0.25 μm. On the other hand, the sizes were ranging from 0.25 to 1.6 μm with peak distribution of about 0.7 μm, for Fig 2c. Finally, for Fig. 2d, the AuNPs distribution is ranging from 0.25 to 1.4 μm and the peak distribution is about 0.25 m. Finally, in Fig. 2e, the AuNPs distribution is ranging from 0.25 to 0.8 μm and the peak distribution is about 0.15 μm. Figure 3 displays the EDS analysis that indicates the existence of AuNPs on the n-type PS surface.

### Raman Measurements

**Fig. 4:**
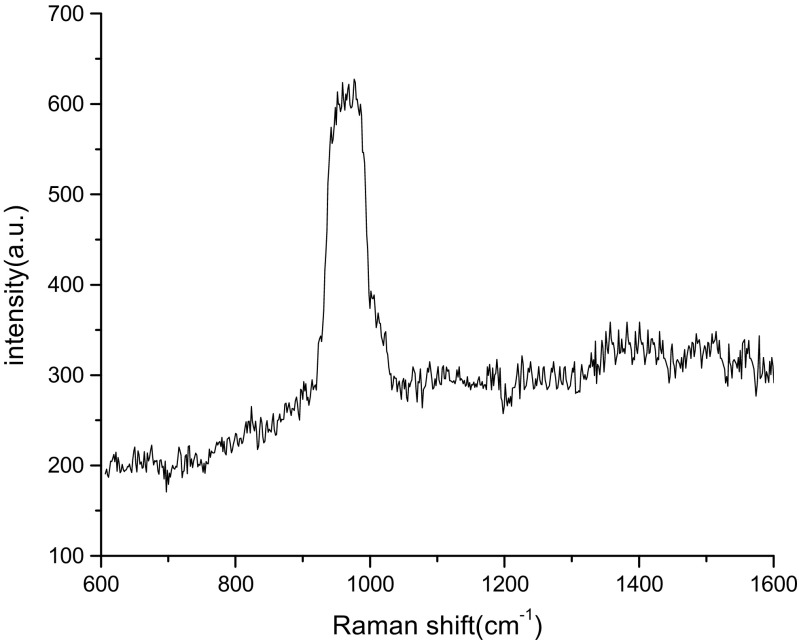
Raman shift of Cy dye prepared at a concentration of 10^−4^ for bare PS (reference)

**Fig. 5:**
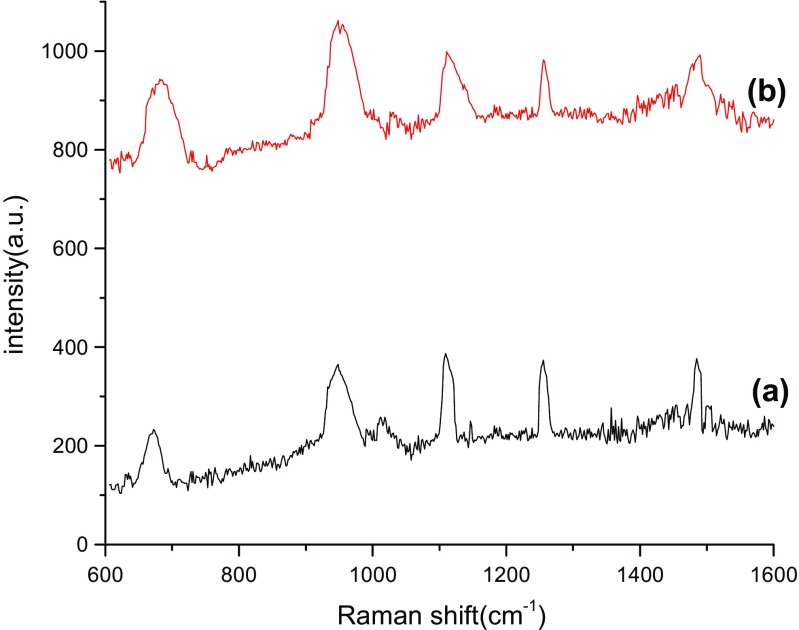
Raman spectra of PS/AuNPs hybrid structure samples deposited with HAuCl_4_ at (**a**) 10^−2^ M and (**b**) 10^−2^diluted in HF for 10^−10^ M of Cy molecules

**Fig. 6:**
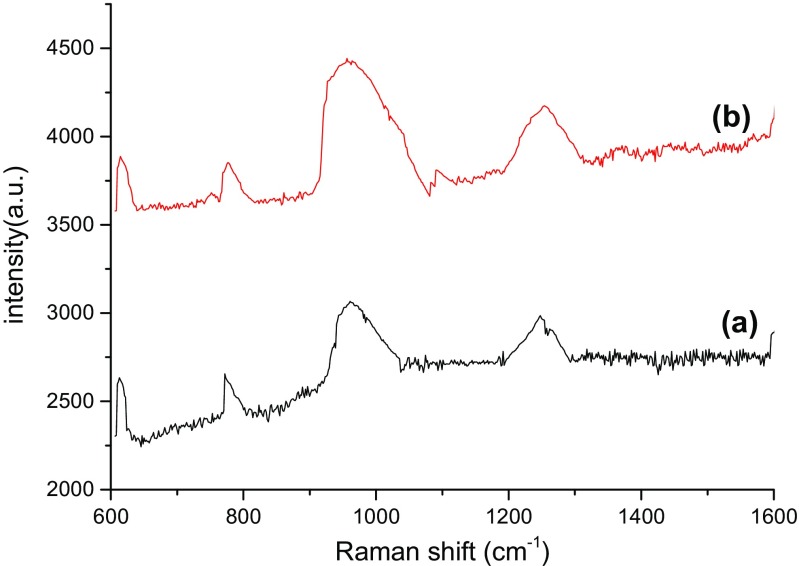
Raman spectra of PS/AuNPs hybrid structure samples deposited with HAuCl_4_ at a) 5 × 10^−3^ M, b) 5 × 10^−3^ diluted in HF for 10^−10^ M of Cy molecules

**Fig. 7:**
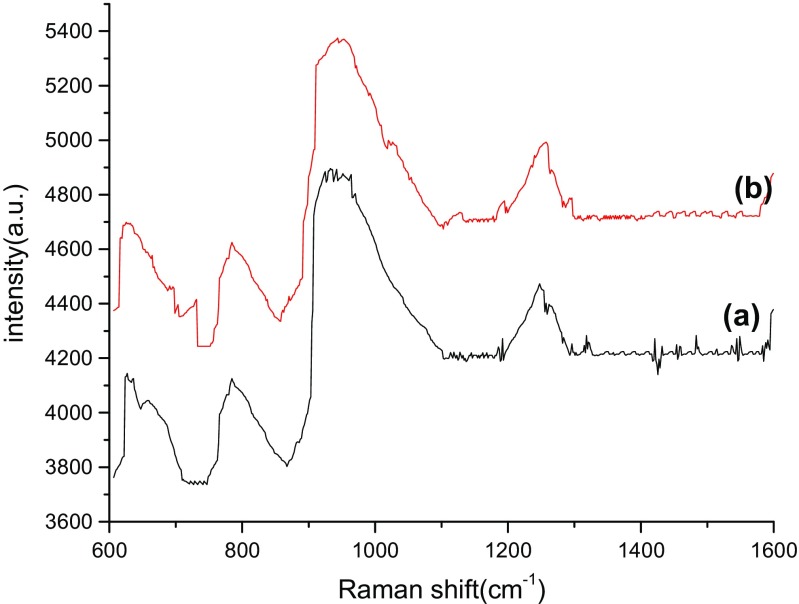
Raman spectra of PS/AuNPs hybrid structure samples deposited with HAuCl_4_ at a) 10^−2^ M, b) 10^−2^ diluted in HF for 10^−6^ M of Cy molecules.

**Fig. 8:**
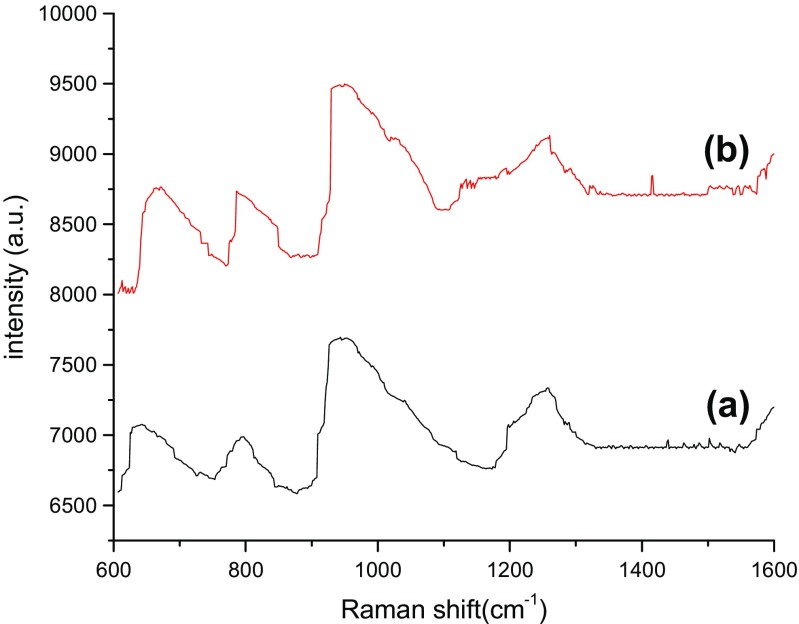
Raman spectra of PS/AuNPs hybrid structure samples deposited with HAuCl_4_ at a) 5 × 10^−3^ M, b) 5 × 10^−3^diluted in HF for 10^−6^ M of Cy molecules

The detection parameters of Cy dye at different concentrations from 10^−4^ to 10^−10^ M was carried out by recording Raman’s signal intensity, Raman shift, assignment bonds, and the Raman enhancement factors as shown in Tables 2, 3, 4, and 5, respectively. Raman spectra of bare PS substrates was tested at a high concentration of dye molecules of about 10^−4^ M (Fig. 6), while for the PS/AuNPs hybrid structure, SERS spectra was carried out at different values of dye concentrations 10^−6^ and 10^−10^ M in Figs. 4, 5, 6, 7, and 8.

**Table 2:** Raman shift, Raman intensity, and assignments of 10^−10^ M Cy molecules for PS/AuNPs hybrid structure samples deposited with a) 10^−2^ M and b) 10^−2^ M of HAuCl_4_ diluted in HF

Assignment	a) 10^−2^ M		b)10^−2^ M of HAuCl_4_ diluted in HF	
	Wave numbers (cm^−1^)	Raman intensity (a.u.)	Wave numbers (cm^−1^)	Raman intensity (a.u.)
Methine chain	672	233	681	943
C–H aromatic ring bending	948	365	948	1062
C–H in plane bending	1109	387	1121	1012
C–C stretching	1255	373	1261	993
CH3 deformation	1485	377	1490	989
	–	EF = 0.63 × 10^6^	–	EF = 1.7 × 10^6^

**Table 3:** Raman shift, Raman intensity, and assignments of 10^−10^ M Cy molecules for PS/AuNPs hybrid structure samples deposited with a) 5 × 10^−3^ M and b) 5 × 10^−3^ M of HAuCl_4_ diluted in HF

Assignment	a) 5 × 10^−3^ M		b)5 × 10^−3^ M of HAuCl_4_ diluted in HF	
	Wave numbers (cm^−1^)	Raman intensity (a.u.)	Wave numbers (cm^−1^)	Raman intensity (a.u.)
Methine chain	612	2633	614	3887
C–H deformation	771	2655	776	3853
C–H aromatic ring bending	960	3066	956	4442
Aromatic C–C stretching	1248	2985	1254	4174
C–N stretching	1606	2939	1614	4297
–	–	EF = 5 × 10^6^	–	EF = 7.2 × 10^6^

**Table 4:** Raman shift, Raman intensity, and assignments of 10^−6^ M Cy molecules for PS/AuNPs hybrid structure samples deposited with HAuCl_4_ at a) 10^−2^ M and b) 10^−2^ M diluted in HF

Assignments	a) 10^−2^ M		b) 10^−2^ M diluted in	
	Wave numbers (cm^−1^)	Raman intensity (a.u.)	Wave numbers (cm^−1^)	Raman intensity (a.u.)
Methine chain	627	4144	625	4699
C–H deformation	–	–	784	4625
C–H aromatic ring bending	932	4896	943	5375
C–N stretching	1247	4473	1257	4993
CH3 deformation	1431	4244	1444	4749
C–N stretching	1606	4427	–	–
**–**	**–**	EF = 8 × 10^2^	–	EF = 9 × 10^2^

**Table 5:** Raman shift, Raman intensity, and assignments of 10^−6^ M Cy molecules for PS/AuNPs hybrid structure samples deposited with HAuCl_4_ at a) 5 × 10^−3^ M, b) 5 × 10^−3^diluted in HF

Assignments	a) 5 × 10^−3^ M		b) 5 × 10^−3^ diluted in HF	
	Wave numbers (cm^−1^)	Raman intensity (a.u.)	Wave numbers (cm^−1^)	Raman intensity (a.u.)
Methine chain	642	7067	672	8766
C–H deformation	796	6989	786	8736
C–H aromatic ring bending	943	7695	950	9499
Aromatic C–C stretching	1257	7335	1260	9133
CH3 deformation	–	–	1416	8848
C–N stretching	1608	7241	–	–
–	**–**	EF = 12.5 × 10^2^	–	EF = 14.8 × 10^2^

For bare PS, the Raman shift and Raman spectra show intensity at 976 cm^−1^ and 613 a.u., respectively

The effect of salt concentration and the role of HF acid on the Raman spectra are given in Fig. 5, 6, 7, and 8 for two concentrations of Cy molecules 10^−6^ and 10^−10^ M, respectively. The Raman intensity and hence the enhancement factors were varied according to the formed gold nanoparticles. This is probably due to the plasmonic effect of AuNPs and the existence of “hot spot” regions at which the local electric field is extremely intense in these regions and high values of EF, which can be defined as the ratio of the radiation intensities of SERS and normal Raman scattering per molecule [9]. The values of the enhancement factor of Raman’s signals are calculated using the following equation [15]:

6 $$ \mathrm{EF}=\left({I}_{\mathrm{SERS}}/{I}_{\mathrm{Raman}}\right)x\ \left({M}_{\mathrm{b}}/{M}_{\mathrm{ads}.}\right) $$

where *M*_b_ and *M*_ads_ are the value of Cy molecule concentrations of dyes in Raman and SERS samples, respectively. *I*_SERS_ and *I*_Raman_ are the intensities of the SERS and Raman spectrum, respectively.

Thus, the Raman signal at those sites is particularly strong and contributes to the main fraction of the overall intensity. Moreover, the creation of very small gold nanoparticles is one of the effective ways to increase S.S.A. and hence improve the efficiency of the energy transfer from plasmonic nanoparticles to Cy molecules [12, 16].

**Fig. 9:**
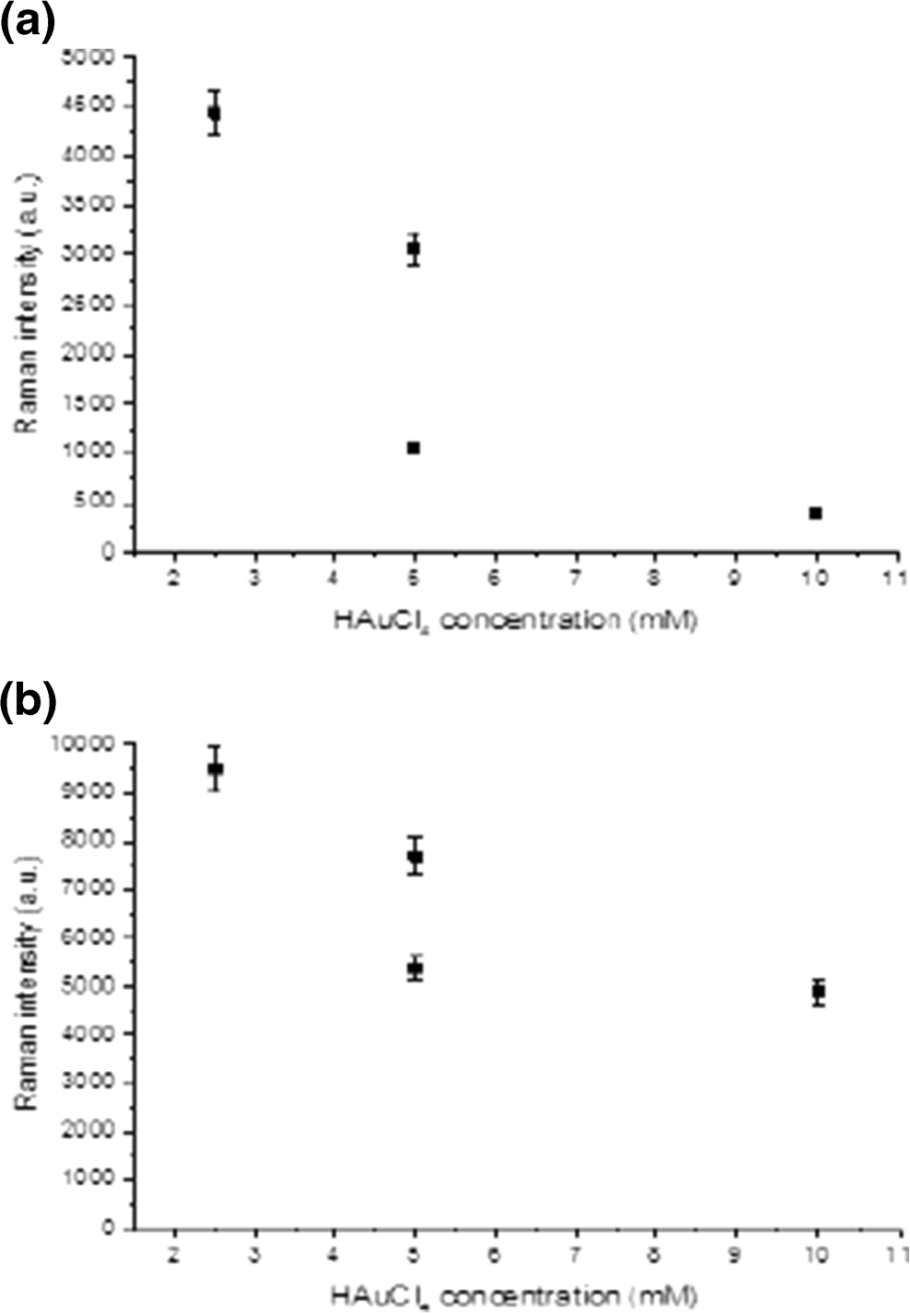
The SERS signal intensity as a function of HAuCl_4_ deposition solution concentration at a)10^−6^ M of CY dye, b)10^−10^ M of Cy molecules

From all these figures and tables at specific dye concentrations, the enhancement factors were increased when adding the HF acid to HAuCL_4_ solution. The best enhancement factors and hence best toxic material detection process obtained from the HAuCl_4_ concentration of the 10^−2^ M with 24% of HF acid. Figure 9a, b shows the variation of SERS signals intensity as a function of the concentration of HAuCl_4_ deposition solution with error bars of about (5%). This was done for two concentrations of Cy molecules of 10^−6^, 10^−10^ M for HAuCl_4_ concentrations of about 10^−2^ M to 5 × 10^−3^ M diluted in HF. Because of diluting the salt solution, the values of Raman’s peaks were increased from 4144 to 9499 a.u. for 10^−6^ M of Cy molecules. While for 10^−10^ M of Cy molecules, the values of Raman’s peaks were increased from 233 to 4442 a.u.

## Conclusion

In this paper, we reported a simple, cost-effective and quick process for the preparation of high sensitive PS/AuNPs Raman active substrates for the chemical sensing process of low concentrations of cyanine molecules. Our outcomes open the possibility of fabricating nano-structured substrates by simple preparation procedures with a high reproducible form, acceptable uniformity, and suitability for numerous applications where the samples need to be analyzed in aquatic solutions. The tunable fabrication of AuNPs (sizes and S.S.A.) over the porous layer will control the chemical detection process. Decreasing the concentration of HAuCl4 in the deposition solution has improved the detection process for the hybrid structure.

## References

[1] Hosny M, Wissem D (2014). Haddadi Ikbel and Ezzaouia Hatem, Influence of gold nanoparticles deposition on porous silicon properties. Sens Transducers.

[2] Dhanekar S, Jain S (2013). Porous silicon biosensor: current status. Biosens Bioelectron.

[3] Kumar Pushpendra, Huber Patrick (2007). Effect of Etching Parameter on Pore Size and Porosity of Electrochemically Formed Nanoporous Silicon. Journal of Nanomaterials.

[4] Herrera GM, Padilla AC, Hernandez-Rivera SP (2013). Surface enhanced Raman scattering (SERS) studies of gold and silver nanoparticles prepared by laser ablation. Nano.

[5] Zhang Y, Chu W, Foroushani AD, Wang H, Li D, Liu J, Barrow CJ, Wang X, Yang W (2014). New gold nanostructures for sensor applications: a review. Materials.

[6] Fernando NT (2011) Novel near-infrared cyanine dyes for fluorescence imaging in biological systems, Dissertation, Georgia state university. http://scholarworks.gsu.edu/chemistry_diss/57

[7] Dempster DN, Morrow T, Rankin R, Thompson GF, Photochemical characteristics of the mode-locking dyes 1,1′-diethyl-4,4′-carbocyanine iodide (cryptocyanine, DCI) and 1,1′-diethyl-2,2′ dicarbocyanine iodide (DDI), Chem Phys Let 18, p 488–492 (1973) 256, p. 6969–6976 (2010)

[8] Giorgis F, Descrovi E, Chiodoni A, Froner E, Scarpa M, Venturello A, Geobaldo F (2008). Porous silicon as efficient surface enhanced Raman scattering (SERS) substrate. Appl Surf Sci.

[9] Virga A, Rivolo P, Frascella F, Angelini A, Descrovi E, Geobaldo F, Giorgis F (2013). Silver nanoparticles on porous silicon: approaching single molecule detection in resonant SERS regime, Applied Science and Technology Department, Politecnico di Torino. J Phys Chem.

[10] Skoog DA, West DM, Holler FJ, Crouch SR (2004) Fundamentals of analytical chemistry, Thomson, United states of America, eight edition

[11] Hadi HA, Ismail RA, Habubi NF (2013). Fabrication and characterization of porous silicon layer prepared by photo-electrochemical etching in CH3OH: HF solution. Int Lett Chem Phys Astron.

[12] Khajehpour K, Williams T, Bourgeois L, Adelojua S (2012) Gold nanothorns—macroporous silicon hybrid structure: a simple and ultrasensitive platform for SERS, Electronic supplementary material (ESI) for chemical communications this journal is © the Royal Society of Chemistry 10.1039/c2cc17078g 22473307

[13] Alwan AM, Hayder AJ, Jabbar AA (2015). Study on morphological and structural properties of silver plating on laser etched silicon. Surf Coat Technol.

[14] Kalita AK, Karmakar S (2016) Research Paper, Effect on particle size and microstrain due to iron doping on Zno nanoparticle prepared by wet chemical method. 5(2). ISSN No 2277 – 8179

[15] Tao A, Kim F, Hess C, Goldberger J, He R, Sun Y, Xia Y, Yang P (2003) Langmuir-Blodgett silver nanowire monolayers for molecular sensing using surface-enhanced Raman scattering spectroscopy

[16] Caro Carlos, M. Paula, Klippstein Rebecca, Pozo David, P. Ana (2010). Silver Nanoparticles: Sensing and Imaging Applications. Silver Nanoparticles.

